# University of Wooster, Ohio

**Published:** 1871-04

**Authors:** 


### University of Wooster, Ohio.

We are indebted to Dr. S. W. Wetmore, of Buffalo, for Catalogue of the University of Wooster, O., the medical department of which is located in Cleveland, in which institution Dr. Wetmore occupies the Chair of *Descriptive and Surgical Anatomy.* The number of students attending the last Term was 58, of which number 25 were of the Graduating Class.

